# Relationship between scoring systems and inflammatory parameters with mortality in pneumonia: Acute Phase Reactant Index is new index

**DOI:** 10.1590/1806-9282.20250005

**Published:** 2025-08-08

**Authors:** Emine Sarcan, Ahmet Burak Erdem, Sinan Özdemir, Gamze Akkoyun, Melike Ayas, Burak Katipoğlu

**Affiliations:** 1 Ministry of Health, Ankara Etlik City Hospital, Department of Emergency Medicine – Ankara, Turkey.

**Keywords:** Community-acquired pneumonia, C-reactive protein, Procalcitonin, Albumin, Mortality

## Abstract

**OBJECTIVE:**

The aim of this study is to investigate the relationship between risk scores and immune-inflammatory indices with prognosis and mortality in community-acquired pneumonia. Additionally, we aim to examine the association between mortality and the Acute Phase Reactant Index, which we developed using C-reactive protein, procalcitonin, and albumin levels.

**METHODS:**

This retrospective study was conducted with 417 patients diagnosed with community-acquired pneumonia who presented to the emergency department of a tertiary care hospital between 2022 and 2023. The patients’ demographic data, imaging findings, blood parameters, site of care, and 30-day mortality status were recorded. Risk scores, immune-inflammatory indices, and the Acute Phase Reactant Index were calculated. The risk scores and indices were compared with treatment outcomes and 30-day mortality results.

**RESULTS:**

In the receiver operating characteristic analysis, Acute Phase Reactant Index was found to be as effective as other risk scores in predicting mortality. Among them, Acute Phase Reactant Index had the highest area under the curve value. Additionally, in patients transferred from the ward to the ıntensive care unit, when the Acute Phase Reactant Index score cut-off value was set at 1.32, it accurately predicted mortality in 9 out of 13 patients (69.2%).

**CONCLUSION:**

It was determined that Acute Phase Reactant Index is a better predictor of mortality than all other scores and inflammatory indices in community-acquired pneumonia. This suggests that the Acute Phase Reactant Index may be used as a prognostic factor in high-mortality lung diseases such as cancer.

## INTRODUCTION

Community-acquired pneumonia (CAP) continues to be one of the most common reasons for emergency department visits and hospital admissions globally. Although mortality in these patients is frequently unavoidable, the management of pneumonia in the emergency department is critically important^1^.

The Pneumonia Severity Index (PSI) and CURB-65 scores are frequently used to determine the appropriate site of care for pneumonia patients^2^. Additionally, scoring systems such as SMART-COP and SCAP (Severe Community-Acquired Pneumonia score) are recommended as guides for assessing pneumonia severity and making decisions regarding intensive care unit (ICU) admission^3,4^. In recent years, there has been an increase in pneumonia pandemics. This has highlighted the need for simpler and more practical inflammatory markers with high predictive value, in addition to pneumonia scoring systems. Most recently, inflammatory markers such as lymphopenia and C-reactive protein (CRP) have significantly aided physicians in making rapid decisions and determining the site of care in COVID-19 pneumonia^5^. Although studies on hemogram values in CAP are limited, they provide valuable results in terms of mortality. Procalcitonin (PCT), which acts like a cytokine and rises in the early hours of the infectious process, along with CRP, has been used to determine prognosis^6^. However, low levels of CRP have been found to be useful in ruling out severe pneumonia^7^. Hypoalbuminemia is an independent risk factor for the development and prognosis of pneumonia. It can accelerate the process of sepsis and bacteremia in patients^8^.

The aim of this study was to examine the correlation of inflammatory indices and scores in CAP and demonstrate the relationship between the Acute Phase Reactant Index (APhRI) derived from CRP, PCT, and albumin and mortality.

## METHODS

### Study design

This study was planned retrospectively on 2,656 patients diagnosed with CAP who were admitted to the Emergency Department of a tertiary care hospital between November 1, 2022, and May 1, 2023. A total of 1,948 patients over the age of 18, who had a preliminary diagnosis of pneumonia, had no history of trauma, and had not received immunosuppressive therapy in the last month, were included in the study. A total of 1,531 patients were excluded due to missing blood parameters, scoring calculations, or findings of thoracic computed tomography (TCT). A total of 417 patients were included in the study. The study was conducted after receiving approval from the local ethics committee and in accordance with the Declaration of Helsinki. Patient information was obtained from the hospital database. The sociodemographic characteristics of the patients, vital signs, oxygen support, consciousness status, hemogram values, CRP, PCT, albumin, TCT findings, risk scores, immune-inflammatory indices, APhRI, hospitalization, and 30-day mortality were recorded.

### Definition

Patients presenting with fever, cough, dyspnea, sputum production, and deterioration in general condition were considered suspected pneumonia cases. Patients with acute changes in inflammatory markers and TCT reports showing findings such as ground-glass opacity, consolidation, cobblestone appearance, air bronchogram, vascular enlargement, and bronchial changes in the medial and lower segments with a peribronchovascular distribution were included in the study with a diagnosis of pneumonia. The risk scores of these patients were calculated, and the Systemic Immune-Inflammation Index (SII), the Systemic Inflammation Response Index (SIRI), the Pan-Immune-Inflammation Value (PIV), and APhRI were evaluated as described below:

SII=Platelet count×neutrophil count/lymphocyte count.

SIRI=Neutrophil count×monocyte count/lymphocyte count.

PIV=Neutrophil count×platelet count×monocyte/lymphocyte.

APhRI= CRP×PCT/albumin.

### Outcome

The primary outcome of this study is to demonstrate that the risk scores are successful in predicting mortality in CAP, with PSI providing the strongest results. The second outcome is to demonstrate that APhRI, as a new index, may be used to assess pneumonia severity and could be a strong predictor of mortality.

### Statistical analysis

Descriptive statistics were presented as frequencies (percentages) for categorical variables and as means with standard deviations for numerical variables. The normality assumption for numerical variables was evaluated both analytically and graphically. Comparison of laboratory data in paired groups was performed using the chi-square test for categorical variables and the ındependent samples t-test or Mann-Whitney U test for numerical variables. The diagnostic ability of laboratory parameters with statistically significant p-values from group comparison tests was assessed using the area under the receiver operating characteristic (ROC) curve. Cut-off values for potential biomarkers were determined using the Youden Index and diagnostic performance metrics, with sensitivity, specificity, and corresponding 95% confidence intervals calculated. Logistic regression analysis was performed for independent risk factors for mortality. Parameters were selected stepwise. For multicollinearity problems, those with variance ınflation factor (VIF) >3 and parameters with high correlation were removed from the model. Statistical analyses were conducted using the Statistical Package for Social Sciences (SPSS, Version 27, SPSS Inc., Chicago, IL) software, and a p-value of <0.05 was considered statistically significant.

## RESULTS

A total of 417 patients were included in the study. There were no statistically significant differences in sociodemographic data, vital signs, and patient outcomes, between the mortality and nonmortality groups (Table 1).

**Table 1:** Sociodemographic data.

		Total (n=417)	Death (n=58)	Alive (n=359)	p-value*
Age (median/IQR)		74 (65–83)	77 (69–85)	74 (64–82)	0.051
Gender	Male (n, %)	252 (60.4%)	48 (82.8%)	204 (56.8%)	<0.001
	Female (n, %)	165 (39.6%)	10 (17.2%)	155 (43.2%)	
None (comorbidite)		83 (19.7%)	7 (12%)	69 (19.2%)	0.002
Malignancy		27 (6.5%)	9 (15.5%)	18 (5%)	
Hepatic diseases		3 (0.7%)	0	3 (0.8%)	
CVD+CVA		211 (50.6%)	25 (43.1%)	186 (51.8%)	
Malignancy+CVD+CVA		28 (6.7%)	9 (15.5%)	19 (5.3%)	
Malignancy+CVD+CVA+RD		5 (1.2%)	2 (3.4%)	3 (0.8%)	
Malignancy+RD		2 (0.5%)	0	2 (0.6%)	
CVD+CVA+RD		29 (7.2%)	5 (8.6%)	24 (6.7%)	
Nursing home resident		56 (13.4%)	10 (17.2%)	46 (12.8%)	0.359
On oxygen support		266 (63.8%)	53 (91.4%)	213 (59.3%)	<0.001
**Vital sign (median/IQR)**					
Temperature		37 (36.4–37.5)	37 (36–37.5)	37 (36.4–37.6)	0.250
SBP (mmHg)		120 (108–137)	110 (92–130)	120 (110–138)	<0.001
DBP (mmHg)		70 (61–80)	65 (57–77)	70 (62–80)	0.012
RR/minute		20 (18–22)	23 (20–28)	20 (18–22)	<0.001
Pulse/minute		95 (82–111)	109 (89–120)	95 (82–110)	0.008
Temperature (°C)		88 (83–93)	85 (80–88)	88 (85–93)	<0.001
SBP (mmHg)		113 (31.9%)	46 (79.3%)	87 (24.2%)	<0.001
Patient outcome (n, %)	Outpatient	110 (26.4%)	0	110	<0.001
	Inpatient	130 (31.2%)	3 (5.2%)	127 (35.4%)	
	ICU	146 (35%)	42 (72.4%)	104 (29%)	
	Transfer	31 (7.4%)	13 (22.4%)	18 (5%)	

* Chi-square/Mann-Whitney U test; IQR: ınterquartile range; CVD: cardiovascular disease; CVA: cerebrovascular accident; RD: renal diseases; SBP: systolic blood pressure; DBP: diastolic blood pressure; RR: respiratory rate; ICU: ıntensive care unit admission.

In the univariate analysis of pairwise comparisons, lymphocytes, BUN, creatinine, albumin, CRP, and PCT were found to be statistically different between the groups. In the multivariate regression analysis, AST, CRP, albumin, PCT, and lactate were identified as independent risk factors for mortality (Table 2). In the multivariate analysis, AST, CRP, albumin, PCT, and lactate were identified as independent risk factors for mortality. When parameters with correlations were excluded based on VIF >3, collinearity tolerance, and condition ındex, only CRP, albumin, and PCT were determined to be independent risk factors. The accuracy of the model consisting of CRP, albumin, and PCT was determined to be 89%, with a Cox & Snell R square of 0.225 and a Nagelkerke R square of 0.406. APhRI, derived from the CRP×PCT/albumin formula, was found to be statistically significant in both univariate and multivariate analyses (Table 2).

**Table 2:** Laboratory measurements.

	Univariate analysis			Multivariate analysis			
	Death (n=58)	Alive (n=359)	p-value*	p-value*	Exp (B)	<95%CI	>95%CI
WBC (10^3^/μL)	9.835 (6.032–11.255)	12.020 (7.795–13.645)	0.903	0.623			
Neutrophil (10^3^/μL)	7.090 (4.310–10.255)	8.460 (6.425–10.835)	0.682	0.657			
Lymphocyte (10^3^/μL)	1.010 (0.242–1.782)	1.140 (0.855–2.040)	**0.015**	0.631			
Monocyte (103/μL)	615 (377–825)	690 (510–960)	0.072				
Hematocrit	35 (31–45)	41 (35–43)	0.104	0.405			
Platelet (10^3^/μL)	195.000 (100.750–404.705)	250.000 (203.500–316.000)	0.874	0.988			
Glucose (mg/dL)	271 (255–306)	305 (267–354)	0.903	0.209			
BUN (mg/dL)	53 (19–131)	43 (28–57)	**<0.001**	0.079			
Creatinine (mg/dL)	1.0 (0.56–2.15)	1.0 (0.83–1.37)	**0.024**	0.398			
AST (U/L)	23 (21–28)	25 (19–35)	0.658	**0.034**	0.97	0.943	0.998
Sodium (mmol/L)	133 (131–136)	134 (131–137)	0.690	0.653			
Albumin	28 (22–36)	36 (32–38)	**<0.001**	**<0.001**	0.786	0.724	0.852
CRP (mg/L)	218 (107–333)	46 (24–100)	**<0.001**	**<0.001**	1.007	1.003	1.011
Procalcitonin (μg/L)	1.47 (0.92–17.5)	0.11 (0.06–0.22)	**<0.001**	**0.005**	1.167	1.047	1.300
Lactate	3.5 (2–7.5)	2.6 (2.2–3.6)	0.225	**0.003**	1.286	1.090	1.517

* Logistic regression analysis, Exp (B): exponentiation of the B coefficient (odds ratio); lower 95%CI: lower bound of the 95% confidence ınterval; upper 95%CI: upper bound of the 95% confidence ınterval; WBC: white blood cell; BUN: blood urea nitrogen; AST: aspartate aminotransferase; CRP: C-reactive protein. The bolded values indicate statistically significant results (p<0.05).

When pneumonia scores, inflammatory indices, and APhRI were evaluated as mortality indicators, CURB-65, PSI, SMART-COP, SCAP, and APhRI were found to be statistically significant between the groups (p<0.001). When the scores and indices were evaluated as indicators of ICU admission, CURB-65, PSI, SMART-COP, SCAP, APhRI, and SII were found to be statistically significant between the groups (p<0.001).

**Figure 1:**
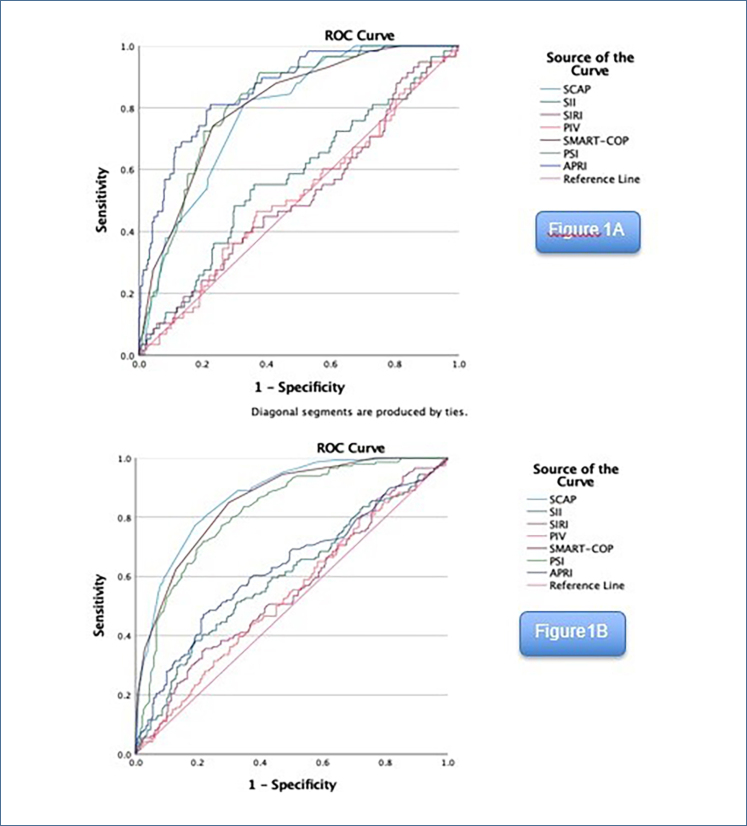
(A) Receiver operating characteristic analysis of pneumonia scores and ındices for mortality. (B) Receiver operating characteristic analysis of pneumonia scores and ındices for ıntensive care unit admission. SCAP: severe community-acquired pneumonia; SII: Systemic Immune-Inflammation Index; SIRI: Systemic Inflammation Response Index; PIV: pan-ımmune-ınflammation value; SMART-COP: systolic blood pressure, multilobar involvement, albumin, respiratory rate, tachycardia, confusion, oxygenation, pH; PSI: Pneumonia Severity Index; APhRI: Acute Phase Reactant Index.

In the ROC analysis, APhRI, CURB-65, PSI, SMART-COP, and SCAP were identified as potential mortality indicators among the scores and indices (0.860, 0.801, 0.818, 0.808, and 0.778). Among them, APhRI had the highest area under the curve (AUC) value (Figure 1A). When the APhRI value of 1.32, obtained from the ROC analysis, was selected as the cut-off, it correctly predicted nine out of 13 patients (69.2%) who progressed from the ward to the ICU and had a fatal outcome. Additionally, an ROC analysis was conducted for ICU admission in relation to pneumonia scores and indices. SII, APhRI, CURB-65, PSI, SMART-COP, and SCAP were identified as potential indicators for ICU admission (0.599, 0.628, 0.841, 0.821, 0.856, and 0.872). Among them, SCAP had the highest AUC value (Figure 1B). When the APhRI score with a cut-off value of 1.26, obtained from the ROC analysis, was selected, it correctly predicted ICU admission in 15 out of 31 patients (48.4%) who progressed from the ward to the ICU.

## DISCUSSION

This study demonstrated the effectiveness of scores and inflammatory indices in determining mortality in patients with CAP. APhRI was superior to other indices and scores in predicting mortality in CAP.

In this study, the gender distribution, age range, comorbidities, mortality rate, and mortality predictions of PSI and CURB-65 were consistent with the literature. SCAP, however, was superior to all other indices and scores in predicting the need for intensive care. The increase in comorbidities with age, along with the more severe course of pneumonia in male patients, reduces the sensitivity and specificity in assessing mortality in CAP and can lead to errors in risk scores. The fact that researchers continue to work on different CAP mortality assessments indicates their desire to reduce error margins in the scores and establish simpler systems^9^. Evaluations made using hemogram parameters remain controversial in regression analyses. Studies have shown that PCT and CRP improve the diagnostic accuracy of CAP. The fact that PCT is not expected to rise in viral pneumonia is important for diagnosis. PCT has been shown to be the most aligned marker with the PSI score and has contributed more than leukocytes and CRP. A review concluded that PCT and CRP are more useful in guiding antibiotic therapy decisions rather than for diagnosis and prognosis^10–12^. Hypoalbuminemia worsens the course of CAP or other critical illnesses due to its roles in maintaining osmotic pressure, transporting various compounds, carrying antibiotics, its antioxidant properties, binding bacterial products, and its immunomodulatory effects^13^.

In pairwise comparisons, there was concordance between the risk scores and APhRI values for mortality. The negative predictive value was near perfect. In comparisons for making ICU admission decisions, APhRI again showed concordance with the risk scores but the SCAP score had the highest AUC value. Additionally, APhRI predicted the mortality of two-thirds of the patients who were transferred from the ward to the ICU and subsequently died, while they were still in the emergency department. It also identified that half of these patients needed to be monitored in the ICU. Although APhRI may not predict ICU admission as effectively as the scores, its strong ability to predict mortality suggests that patients with high APhRI values should be monitored in the ICU. In contrast, the scores significantly underestimated the mortality rate of these patients. When evaluating the results of the study, it was found that, according to the univariate regression analysis, lymphocytes, urea, creatinine, albumin, CRP, and PCT were statistically different between the groups. In the second stage, a multivariate regression analysis was performed. AST, CRP, albumin, PCT, and lactate were found to be significant in terms of mortality. In the third stage, when parameters with multicollinearity issues were excluded, albumin, CRP, and PCT were identified as independent risk factors. Based on clinical studies and the statistical results we obtained, APhRI was ultimately derived by dividing the product of PCT and CRP by albumin. The reason for dividing by albumin is its role as a negative acute phase reactant. Infections are the most common cause of hypoalbuminemia in acute phase reactions. Albumin is one of the leading products in antioxidant processes. Although the liver increases albumin synthesis to manage the antioxidant process, the breakdown caused by inflammatory events can be greater. This contributes to the development of hypoalbuminemia. Additionally, a close relationship has been identified between the increase in CRP and hypoalbuminemia. It is used in the SMART-COP score, and its accuracy has been established^9^. A study found that the SOFA score and the CRP-albumin ratio were significant for 28-day mortality in elderly patients admitted to the ICU. No significant relationship was found between inflammatory indices and death in these patients. Only SIRI was found to be high^14^. These indices have shown more successful results in cancer patients and in pneumonia associated with stroke^14,15^. In this study, SII was found to be significant in patients admitted to the ICU. However, it was not as sensitive as APhRI. In a study related to chronic obstructive pulmonary disease, SII was found to be associated with mortality. The increase in SII paralleled the deterioration of respiratory function^16^. This mechanism may explain the relationship between SII and ICU admission in CAP patients in this study, as a result of the deterioration in their respiratory function.

### Limitations

One important limitation of the study was its retrospective nature. We were unable to access some data, which reduced our sample size. The absence of sputum cultures prevented the etiological differentiation of pneumonia. Stronger results could have been obtained by comparing emergency department data with other data from the patient's hospitalization process. Statistical regression analyses and the APhRI we developed were the strongest aspects of the study.

## CONCLUSION

AFRI was identified as the best predictor of mortality compared to all other scores and inflammatory indices. APhRI can be used for rapid decision-making in mortality prediction in CAP. APhRI can add a new index to the literature with its validity in prospective studies.

## Data Availability

The datasets generated and/or analyzed during the current study are available from the corresponding author upon reasonable request.
